# Molecular docking analysis of novel quercetin derivatives for combating SARS-CoV-2

**DOI:** 10.6026/97320630019178

**Published:** 2023-02-28

**Authors:** Rageh K. Hussein, Mohammad Marashdeh, Ahmed M El-Khayatt

**Affiliations:** 1 Department of Physics, College of Science, Imam Mohammad Ibn Saud Islamic University (IMSIU), Riyadh, Saudi Arabia

**Keywords:** Quercetin-based derivatives, SARS-CoV-2, ADMET prediction, molecular docking

## Abstract

Quercetin belongs to the flavonoid family, which is one of the most frequent types of plant phenolics. This flavonoid compound is a natural substance having a number of pharmacological effects, including anticancer and antioxidant capabilities, as well as being a strong inhibitor of various toxicologically important enzymes. We discuss the potential of newly recently synthesized quercetin-based derivatives to inhibit SARS-CoV-2 protein. ADMET analysis indicated that all of the studied compounds had low toxicities and good absorption and solubility properties. The molecular docking results revealed that the propensity for binding to SARS-CoV-2 main protease is extraordinary. The results are remarkable not only for the binding energy values, which outperform several compounds in prior studies, but also for the number of hydrogen bonds formed. Compound 7a was capable of forming 10 strong hydrogen bonds as well as interact to the protein receptor with a binding energy of -7.79 kcal/mol. Therefore, these compounds should be highlighted in further experimental studies in the context of treating SARS-CoV-2 infection and its effects.

## Background:

Flavonoids are naturally occurring compounds with an aromatic rings and at least one hydroxyl group. These flavonoid components have been identified as a promising and attractive candidate for pharmacology and therapeutic uses [1, 2]. Quercetin is a kind of a flavonoid aglycone that is found in approximately all edible fruit and vegetables. It is a versatile antioxidant known to possess protective abilities against tissue injury induced by various drug toxicities [3]. Compositions of quercetin have attracted interest in recent years due to their broad pharmacological profile. Many Scientific researches have been designed to evaluate the effect of quercetin derivatives in the treatment of SARS-CoV-2. Quercetin has been found to reduce SARS-Cov-2 protease activity by binding to it via the hydroxyl groups [4, 5, 6]. Recently, the synthesis of hybrid compounds involving pharmacophore fragments and their assessment as potent therapeutic candidates has been steadily increasing. The acylation reactions are the most often used approach for producing quercetin derivatives; another frequently used technique is C-amino alkylation via the Mannich reaction [7, 8]. Bioinformatics, an integrated field of science and information technology, produces very relevant results when analyzing biological systems. Computational techniques are predictive strategies for describing and characterizing chemical compounds prior to performing scientific experimental studies [9]. Molecular docking has a lot of value nowadays as one of the computational techniques to generate significant results in fundamental biological research. While ADMET prediction models are used to compute the properties of chemical substances in order to reflect their acceptability for human use[10]. The current study investigated the interactions of five new quercetin derivatives with 6LU7, the main protease of SARS-CoV-2. A molecular docking study was carried out to identify the best poses and associated binding affinities for the proposed compounds in the cavity binding site of the target protein.

## Material and Methods:

## Experimental Details

**Figure 1:**
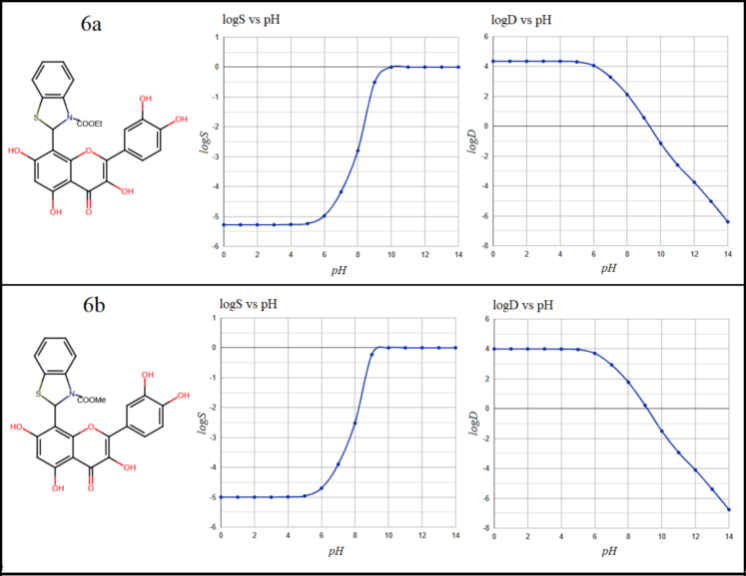
The molecular structure, along with the solubility logS and dispersion coefficient (log D), of compounds 6a and 6b as a result of pH fluctuation.

The studied molecules were recently synthesized by Desislava Kirkova et al. The published work includes the synthesizing procedure as well as an evaluation of radical scavenging activity and spectroscopic characterization data in the supplementary attachments [11]. The five compounds produced by this effort were symbolized as 6a, 6b, 7a, 8a, 8b, and their molecular structures are represented in Figures 1 - Figure3.

## Physico-chemical properties:

The physicochemical properties which describe the Pharmacokinetics behaviour such as the partition coefficient n-octanol/water (LogP), distribution coefficient (logD) and The aqueous solubility (logs) were predicted by using "ChemAxon's Calculator Plugins" Web server (https://disco.chemaxon.com/ calculators/demo/plugins/)

## Protein Preparation:

The protein PDB ID: 6LU7 has been identified as the principal protease of SARS-CoV-2, which is necessary for viral replication and hence a major target for inhibitor drugs. The 3D protein crystal structure was obtained from the protein data bank database and prepared using the Autodock 4.2 [12].

##  Molecular Docking:

The molecular docking was conducted using Auto Dock software to identify the best stable pose with the highest negative binding energy value for receptor-ligand interactions. The ligands optimized structures were positioned in a grid box with dimensions of 40 x 40 x 40 and a spacing of 0.375 generated by the Auto Dock tool. The genetic algorithm (GA) parameters were set at 100 GA runs, while the other parameters were left at their default values. Discovery Studio 4.0 was used to visualize the receptor-ligand interactions [13].

## Result and Discussion:

## ADMET evaluation:

**Figure 2:**
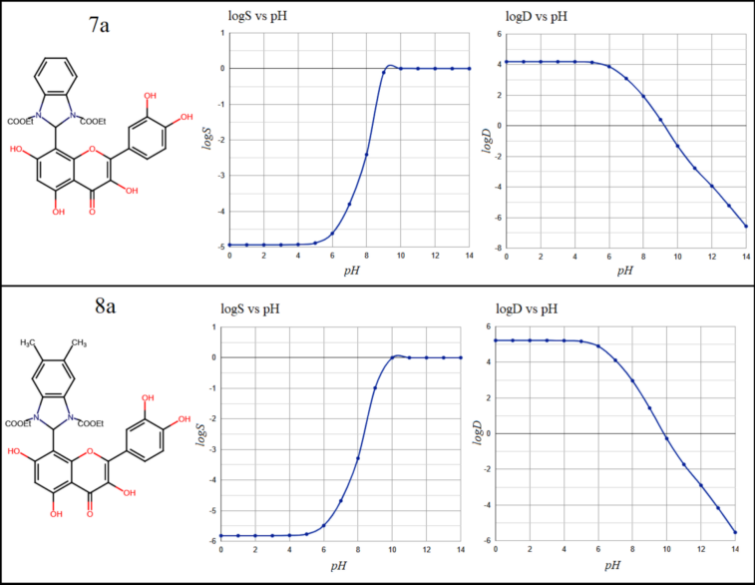
The molecular structure, along with the solubility logS and dispersion coefficient (log D), of compounds 7a and 8a as a result of pH fluctuation.

**Figure 3:**
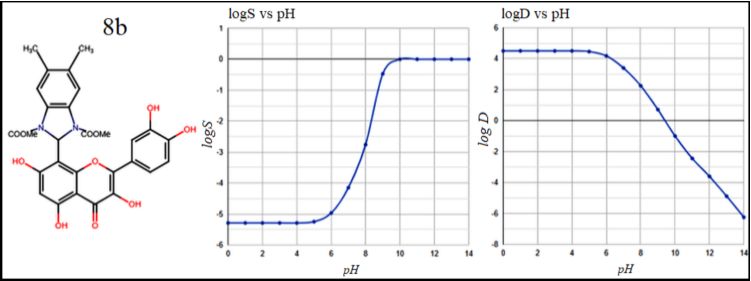
The molecular structure, along with the solubility logS and dispersion coefficient (log D), of compounds 8b as a result of pH fluctuation.

The five compounds were evaluated for drug-likeness, toxicity, and other pharmacokinetic characteristics. In general, compounds that are not particularly lipophilic have a higher bioavailability and better absorption in the intestine. The n-octanol/water partition coefficient (logP), n-octanol/water distribution coefficient (log D) and the aqueous solubility (logS) are crucial parameters in drug evaluation since they are frequently used to determine the bioavailability and toxicity level of the chemical compounds[14] . The criterion for approving new oral drugs to not lipophilic and so has a higher bioavailability is in the range logP < 5 [15]. In Tabl e 1, the calculated LogP values were found ≤ 4.40 which reflect a low lipophilic character of the stated compounds. Compounds with logD values < 3 at physiological pH (7.19) have been shown to have high intestinal absorption. As shown in Figure 1,Figure 2, Figure 3, LogD values ranging from 4.0 to 5.1 suggest that the compounds exceed the optimal lipophilicity limit. The values of logD at pH 7.19 in the order of 3.10 and these values decrease as the pH levels increase, indicating that lipophilicity tends to diminish with increasing pH. The high solubility was found for 7a compound (logs = -4.95) while the compound 8a achieved the lowest solubility (logs = -5.8), as indicated in Figures 1 ,Figure 2, Figure 3. Therefore, the studied compounds were classified as weakly soluble based on their predicted logS values [16]. However, in a similar manner to the distribution coefficient results, increasing the pH improves solubility, with an average value logS ≈ - 4 at physiological pH =7.19. Table 1 also includes a list of other important pharmacokinetic properties. Drug permeability to the blood-brain barrier (BBB) is an important pharmacokinetic factor. All of the compounds tested can cross through the BBB with a probability greater than 0.5 [17]. The Human intestinal absorbance probability (HIA) of the ligands evaluated was > 0.65, indicating good absorption efficiency. Non inhibition of Cytochrome P450 and its major isoforms is required for safe pharmacokinetic interaction of drugs. The toxicity of a compound decreases as the Rat Acute Toxicity (LD50) value increases; a low toxic drug has a high LD50 value. The calculated LD50 values classify the compounds in the safe category [18].

## Molecular docking:

**Figure 4:**
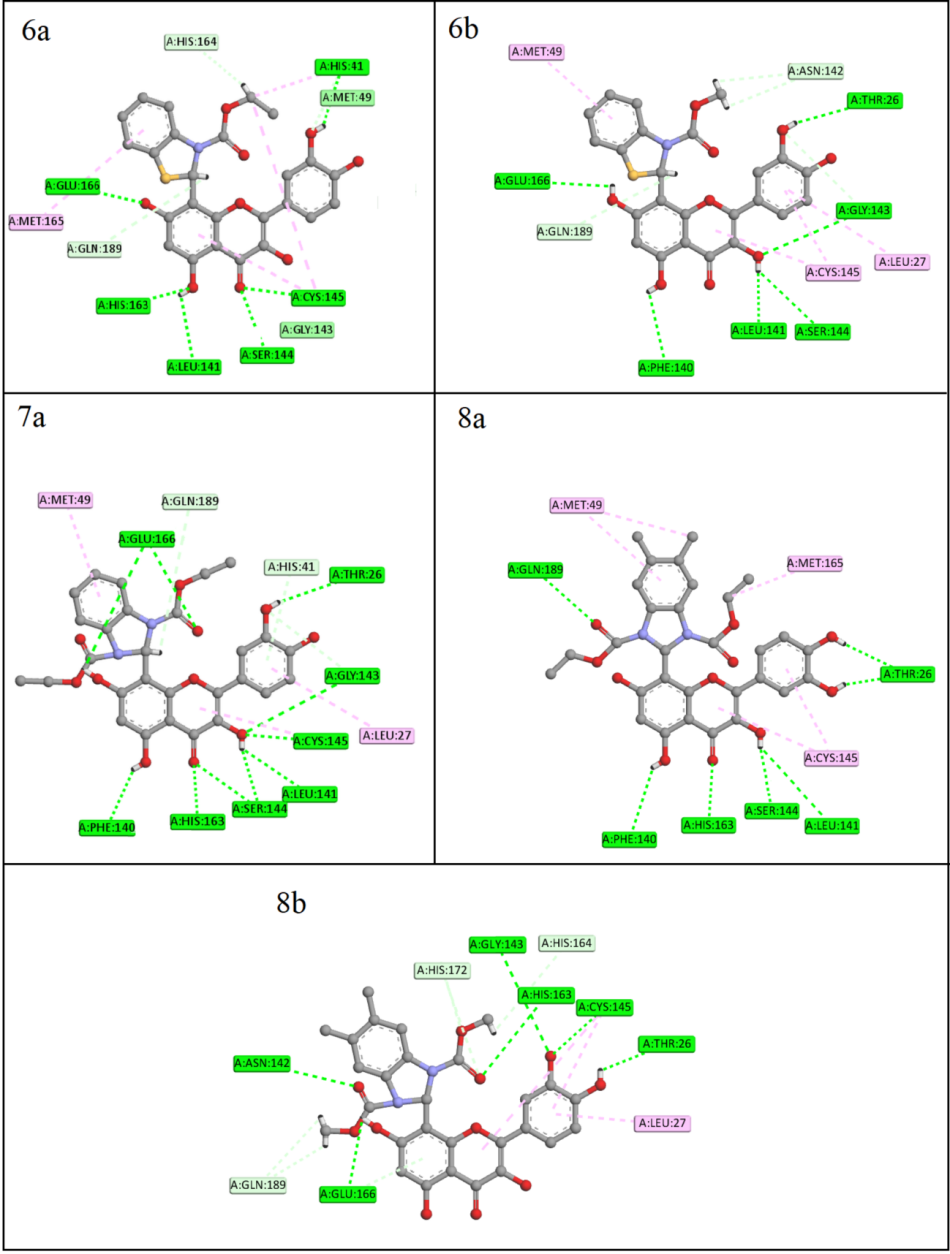
2D representation of the interaction of the five ligands within the active binding sites of the SARS- CoV-2 main protease 6LU7

The molecular docking investigations were run for each of the five ligands within the active site the 6lu7 main protease of SARS-CoV-2. The strength of the protein-ligand interaction is determined by three major parameters derived from molecular docking results. The three parameters are the binding energy, inhibition constant and number of hydrogen bonds. AS reducing the binding energy, lowering the inhibition constant, and increasing the number of hydrogen bonds strengthens the interaction, and vice versa. The docking results are listed in Table 2, and the 2D patterns for the most stable interaction are illustrated in Figure 4. In comparison to previous studies on the same receptor protein, the five ligands have a good docking score, with strong binding energies ranging from -7.58 to -7.79 kcal/mol [19]. The lowest binding energy was obtained by the 7a ligand. Furthermore, the target protein's strong interaction with the ligands was validated by the obviously low inhibition constant values. The most remarkable finding is the number of hydrogen bonds formed. Ligand 7a demonstrated superior inhibitory capability for SARS- CoV-2 main protease through ten conventional hydrogen bonds. While the other ligands 6a, 6b, and 8b formed six hydrogen bonds, and ligand 8a formed seven. Figure 1 shows that the ligands successfully formed H-bonds with the important residues of the main protease binding pocket. The majority of the amino acid residues involved in the interaction with the ligands is Glu166, Phe140, and Gln189, Cys145, Ser144 and Gly143. These are the same majority amino acid residues that were included in the previously reported work "Structure of Mprofrom SARS-CoV-2 and discovery of its inhibitors," which is available on the PROTEIN DATA BANK website, the source to obtain the pdb file of 6lu7 main protease.

## Conclusion:

This is an in silico study that was utilized to evaluate the therapeutic characteristics of five recently synthesized quercetin derivatives against the main SARS-CoV-2 target protein. Compared to prior investigations, the compounds displayed a high binding affinity for the 6lu7 main protease of SARS-CoV-2. This was proven by the high binding energies and the number of conventional hydrogen bonds formed. The obtained ADMET value indicated a profile with acceptable toxicity and absorption properties. The findings could serve as a springboard for more in vitro and in vivo investigation on these compounds in order to develop potentially potent SARS-CoV-2 inhibitors.

## Figures and Tables

**Table 1:** The predicted ADMET profile of the proposed compounds including physicochemical and pharmacokinetic properties.

	**6a**		**6b**		**7a**		**8a**		**8b**	
**Model**	**Result**	**Pro-bability**	**Result**	**Pro-bability**	**Result**	**Pro-bability**	**Result**	**Pro-bability**	**Result**	**Pro-bability**
Blood-Brain Barrie	BBB	0.604	BBB	0.706	BBB	0.54	BBB	0.5	BBB	0.534
Human Intestinal Absorption	HIA+	0.852	HIA+	0.656	HIA+	0.874	HIA+	0.863	HIA+	0.763
P-glycoprotein Substrate	Non-substrate	0.565	Non-substrate	0.607	Non-substrate	0.654	Substrate	0.647	Substrate	0.56
P-glycoprotein Inhibitor	Non-inhibitor	0.834	Non-inhibitor	0.846	Non-inhibitor	0.834	Non-inhibitor	0.77	Non-inhibitor	0.736
CYP450 2C9 Substrate	Non-substrate	0.738	Non-substrate	0.707	Non-substrate	0.829	Non-substrate	0.817	Non-substrate	0.785
CYP450 2D6 Substrate	Non-substrate	0.824	Non-substrate	0.823	Non-substrate	0.861	Non-substrate	0.853	Non-substrate	0.849
CYP450 3A4 Substrate	Non-substrate	0.539	Non-substrate	0.518	Non-substrate	0.554	Non-substrate	0.518	Substrate	0.515
CYP450 1A2 Inhibitor	Non-inhibitor	0.584	Non-inhibitor	0.584	Non-inhibitor	0.711	Non-inhibitor	0.71	Non-inhibitor	0.771
CYP450 2C9 Inhibitor	Non-inhibitor	0.574	Non-inhibitor	0.677	Non-inhibitor	0.731	Non-inhibitor	0.736	Non-inhibitor	0.854
CYP450 2D6 Inhibitor	Non-inhibitor	0.785	Non-inhibitor	0.823	Non-inhibitor	0.812	Non-inhibitor	0.842	Non-inhibitor	0.831
CYP450 2C19 Inhibitor	Inhibitor	0.517	Inhibitor	0.586	Non-inhibitor	0.649	Non-inhibitor	0.705	Non-inhibitor	0.757
CYP450 3A4 Inhibitor	Non-Inhibitor	0.832	Non-Inhibitor	0.804	Non-Inhibitor	0.833	Non-Inhibitor	0.857	Non-Inhibitor	0.88
AMES Toxicity	Non AMES toxic	0.603	Non AMES toxic	0.629	Non AMES toxic	0.708	Non AMES	0.6955	toxic	0.701
Acute Oral Toxicity	III	0.648	III	0.601	III	0.677	III	0.67	III	0.597
Rat Acute Toxicity	2.402	LD50, mol/kg	2.5039	LD50, mol/kg	2.357	LD50, mol/kg	2.4035	LD50, mol/kg	2.5926	LD50, mol/kg

**Table 2:** The the docking results of the proposed ligands with 6lu7 main protease of SARS-CoV-2; binding energy, inhibition constant and number of hydrogen bonds with the interacting residues.

**Compounds**	**Binding Energy (kcal/mol)**	**Inhibition Constant (µM)**	**Number of Hydrogen Bonds**	**Interacting Residues**	**Bond Distance (Å)**
	-7.72	2.18	6	Cys145	3.08
				Ser144	2.87
				Leu141	3.1
6a				His163	2.87
				Glu166	2.67
				His41	2.98
	-7.71	2.24	6	Thr26	2.9
				Gly143	2.91
6b				Leu141	2.56
				Ser144	3
				Phe140	3.33
				Glu166	3.28
	-7.79	1.93	10	Thr26	3.01
				Gly143	2.97
				Cys145	3.15
				Leu141	2.58
7a				Ser144	2.77
				Ser144	2.8
				His163	2.79
				Phe140	2.64
				Glu166	2.96
				Glu166	3.31
	-7.65	2.46	7	Thr26	3.01
				Thr26	2.75
				Leu141	2.85
8a				Ser144	2.72
				His163	2.74
				Phe140	2.66
				Gln189	2.88
	-7.58	2.76	6	Thr26	2.69
				Cys145	3.34
				His163	3.06
8b				Gly143	3.15
				Asn142	2.96
				Glu166	2.75
